# Simultaneously improving the mechanical and electrical properties of poly(vinyl alcohol) composites by high-quality graphitic nanoribbons

**DOI:** 10.1038/s41598-017-17365-3

**Published:** 2017-12-07

**Authors:** Ming Yang, Lin Weng, Hanxing Zhu, Fan Zhang, Tongxiang Fan, Di Zhang

**Affiliations:** 1 State Key Laboratory of Metal Matrix Composites, Shanghai Jiao Tong University, 800 Dongchuan Road, Shanghai, 200240 P.R. China; 2 School of Engineering, Cardiff University, Cardiff, CF24 3AA UK

## Abstract

Although carbon nanotubes (**CNT**s) have shown great potential for enhancing the performance of polymer matrices, their reinforcement role still needs to be further improved. Here we implement a structural modification of multi-walled CNTs (**MWCNT**s) to fully utilize their fascinating mechanical and electrical properties *via* longitudinal splitting of MWCNTs into graphitic nanoribbons (**GNR**s). This nanofiller design strategy is advantageous for surface functionalization, strong interface adhesion as well as boosting the interfacial contact area without losing the intrinsic graphitic structure. The obtained GNRs have planar geometry, quasi-1D structure and high-quality crystallinity, which outperforms their tubular counterparts, delivering a superior load-bearing efficiency and conductive network for realizing a synchronous improvement of the mechanical and electrical properties of a PVA-based composite. Compared to PVA/CNTs, the tensile strength, Young’s modulus and electrical conductivity of the PVA/GNR composite at a filling concentration of 3.6 vol.% approach 119.1 MPa, 5.3 GPa and 2.4 × 10^−4^ S m^−1^, with increases of 17%, 32.5% and 5.9 folds, respectively. The correlated mechanics is further rationalized by finite element analysis, the generalized shear-lag theory and the fracture mechanisms.

## Introduction

Carbon nanotubes (**CNT**s) have long been recognized as ideal reinforcing agents for composites—typically a polymer-based composite—due to the elegant combination of mechanical, electrical and thermal properties coupling with their low density, large aspect ratio and large specific surface area (**SSA**)^1–3^. CNTs have been widely used as fillers to improve the mechanical properties (in terms of strength, stiffness and toughness)^4,5^, electrical conductivity^6^, piezoelectric^7^, dielectric^8,9^, electromagnetic^10^ and thermal^11^ properties of various types of polymer matrices. However, a critical challenge in nanocomposite fabrication by adding CNTs as reinforcement is to maximize the transfer of the excellent properties of individual CNTs to the macroscopic properties of the matrix. Although individual CNTs have exhibited extraordinary properties, their reported reinforcement efficiency is still limited due to their bundle or aggregate forming behavior and the poor wetting and interfacial adhesion with the polymer chains. In this regard, functionalization of CNTs is always prerequisite for achieving their outstanding mechanical, electrical and biological functions and enhancing their dispersion in polymer matrices^3,12^. A large portion of the recent publications on CNTs have focused on enhancing their solubilization and dispersion using non-covalent or covalent functionalization methods^9,13–15^.

On the other hand, the reinforcement role of pristine CNTs (**P-CNT**s), especially multi-walled CNTs (**MWCNT**s) with large diameter, has been restricted by the limited available interfacial contact area with the polymer matrices. MWCNTs are naturally consisted of nested graphene cylinders, where the outermost cylinder shields the internal tubes from the matrix and only the defective outermost walls can interplay with the polymer matrix and carry the load^16–19^. Therefore, the exceptional strength of inner graphene cylinders in MWCNTs is futile, while the inter-tube slip within the concentric nanotube cylinders may lead to a ‘sword-in-sheath’ type failure^17,19^. Actually, MWCNTs can be exfoliated into single or few-layered graphene nanoribbons *via in-situ* unzipping or intercalation-exfoliation approaches^20,21^, which may pave new ways to fully utilize the inner walls and thus maximize their load-bearing ability for polymer composites^19,22–26^. Among those approaches for unwrapping MWCNTs, chemical unzipping using strong oxidants (*e*.*g*. KMnO_4_ and H_2_SO_4_)^19,25–27^ should be the most prevailing one due to the low-cost and high-throughput. Nevertheless, as a typical covalent functionalization method, chemical unzipping is inevitably accompanied by an introduction of structural defects and an ensuing loss of intrinsic physical properties, which will restrain their reinforcing effect. Non-covalent approaches may not destroy the sp^2^-carbon framework of P-CNTs^14,28^. However, the load-transfer efficiency might decrease because the bonding between the wrapping molecules and the nanotube surface is relatively weak. In addition, non-covalent approaches can hardly provide any morphological modification or unzipping for P-CNTs.

For practical applications, high-performance polymers or polymer-based composites with multi-parameter properties are always demanded^25^. It is therefore advantageous to implement configurational modification of MWCNTs without seriously destroying the intrinsic graphitic framework, which has the potential to synchronously improve the mechanical and conductive properties of polymer composites. In our previous research^29^, we have developed a nanofiller design strategy through longitudinal splitting of MWCNTs using HNO_3_ vapor. We have fabricated large amount of high-quality, multi-layered graphitic nanoribbons (**GNR**s) by this facile gas-phase treatment method. The planar geometry, inherent quasi-1D structure, larger interfacial area available together with the minimized structural damage of GNRs are expected to benefit a more significant reinforcing effect than their unmodified counterparts. Herein, we further incorporate these GNRs into a polymer matrix to validate this conjecture by testing both the mechanical properties (strength and Young’s modulus) and electrical conductivity of the composites. We select poly(vinyl alcohol) (**PVA**) here, as it is commonly used as a model polymer matrix for composite studies^30^. PVA is a thermoplastic, water-soluble, hydroxyl-rich and biocompatible polymer, which can form hydrogen bonds with oxygenated surfaces. The nanofillers and PVA are blended *via* a facile solution mixing method. For comparison, refluxed CNTs (**R-CNT**s) are also prepared, and the corresponding PVA-based composites reinforced by GNRs and R-CNTs are denoted as **PVA/GNR**s and **PVA/CNT**s, respectively. The results are compared in order to establish the effect of longitudinal cleavage on the reinforcement role of MWCNTs. Significantly, the mechanics and load-transfer effects are further rationalized by finite element analysis, the generalized shear-lag theory and the fracture behaviors, which suggest that the unique geometric factors of GNRs are conducive to a more effective capability for transmitting stress as well as forming a more significant conducting network in PVA matrix.

## Results and Discussion

### Microstructure and Chemical Properties of GNRs

**Figure 1:**
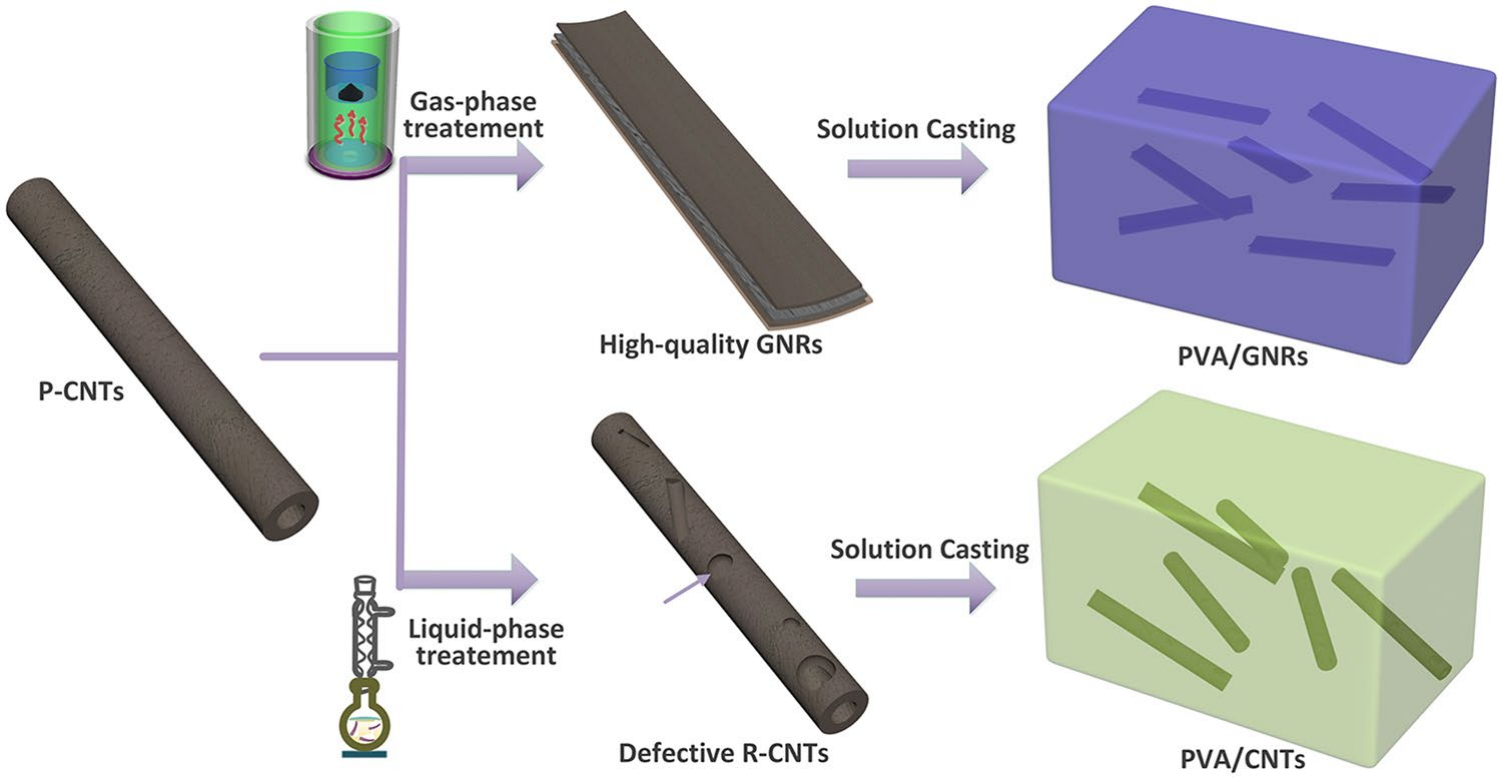
Schematic illustration of the nanofiller design and the fabrication of PVA-based composites.

**Figure 2:**
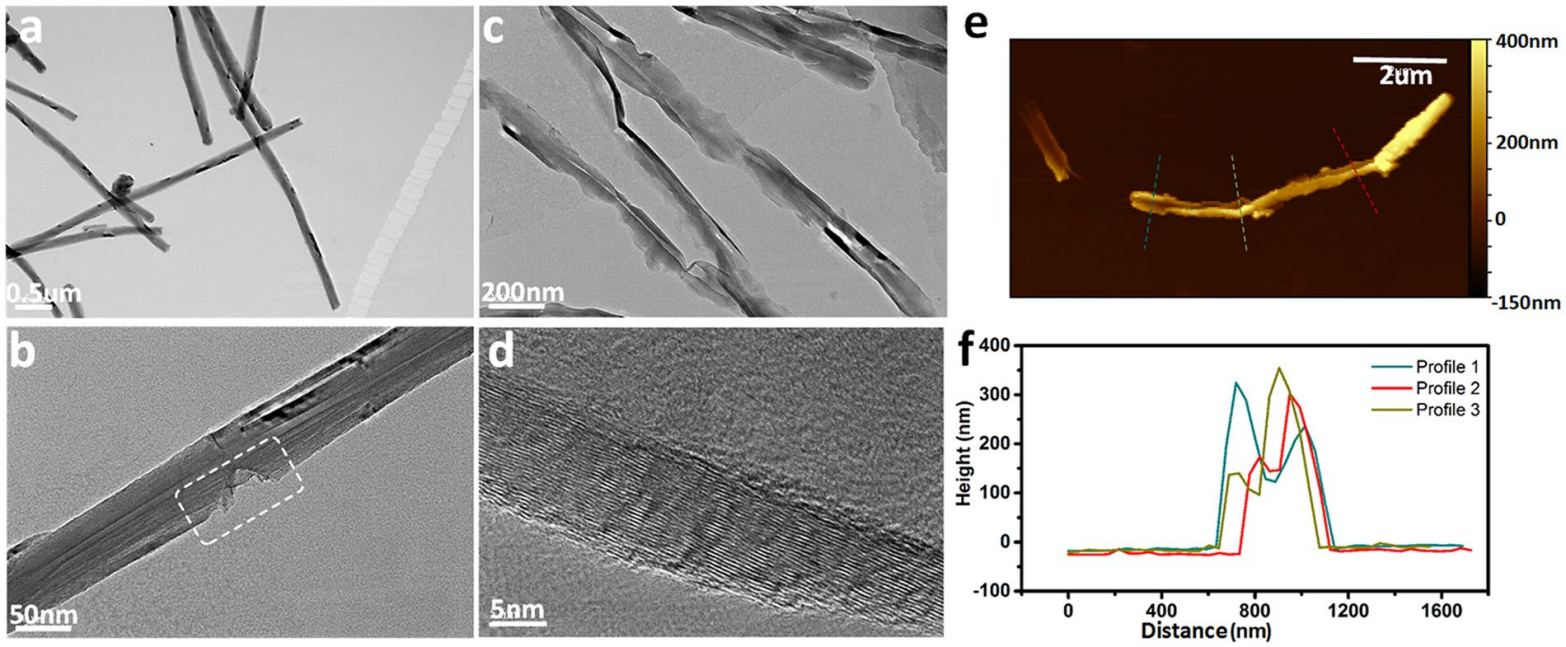
Microstructure of GNRs *via* longitudinal splitting of straight and well-crystallized MWCNTs. (**a–c**) TEM images of P-CNTs, R-CNTs and GNRs, respectively. The box in panel (**b)** indicates groove-like defects of R-CNTs due to chemical attack. (**d**) HRTEM image showing the crystalline side walls of GNRs. (**e**) AFM image of GNRs. (**f**) Height profiles corresponding to the lines shown in panel €.

Figure 1 depicts the schematic illustration of the fabrication of PVA/GNRs and PVA/CNTs. A solution mixing method is employed to uniformly blend the oxygen-functionalized GNRs and R-CNTs within the PVA matrix. The reinforcement role of GNRs are compared with that of R-CNTs to highlight the effect of structural optimization. Figure 2a represents the microstructure of P-CNTs used in this study, which have straight and tubular geometry. It is worth mentioning that unlike the commonly used entangled and curved CNTs, the straight geometry of nanofillers adopted here is helpful for their uniform dispersion in the PVA matrix during processing.

TEM image (Fig. 2b) exhibits that, despite the tubular and linear shape remaining unchanged, R-CNTs have profuse point or groove-like defects, which arise from serious chemical attack by acid reflux^31^. The geometrical transformation from nanotube (P-CNTs) to nanoribbons (GNRs) *via* gas-phase splitting is unambiguously proved by TEM (Fig. 2c,d) and AFM images (Fig. 2e). The opened side-walls of GNRs show crystallized edge with tens of graphene layers, as revealed by HRTEM imaging (Fig. 2d). TEM and AFM images also demonstrate that the as-synthesized GNRs have irregular edges and a length of 4–8 μm, whilst AFM height profiles (Fig. 2f) display a GNR thickness of 20–100 nm. The configuration change is also reflected by the SSA measurements of different samples. Nitrogen cryosorption experiments (Figure S1) reveal that the average SSA of GNRs (35.8 m^2^ g^−1^) is 60% larger than that of R-CNTs (22.6 m^2 ^g^−1^), which is ascribed to the opening, unpeeling and exposure of additional CNT walls.

**Figure 3:**
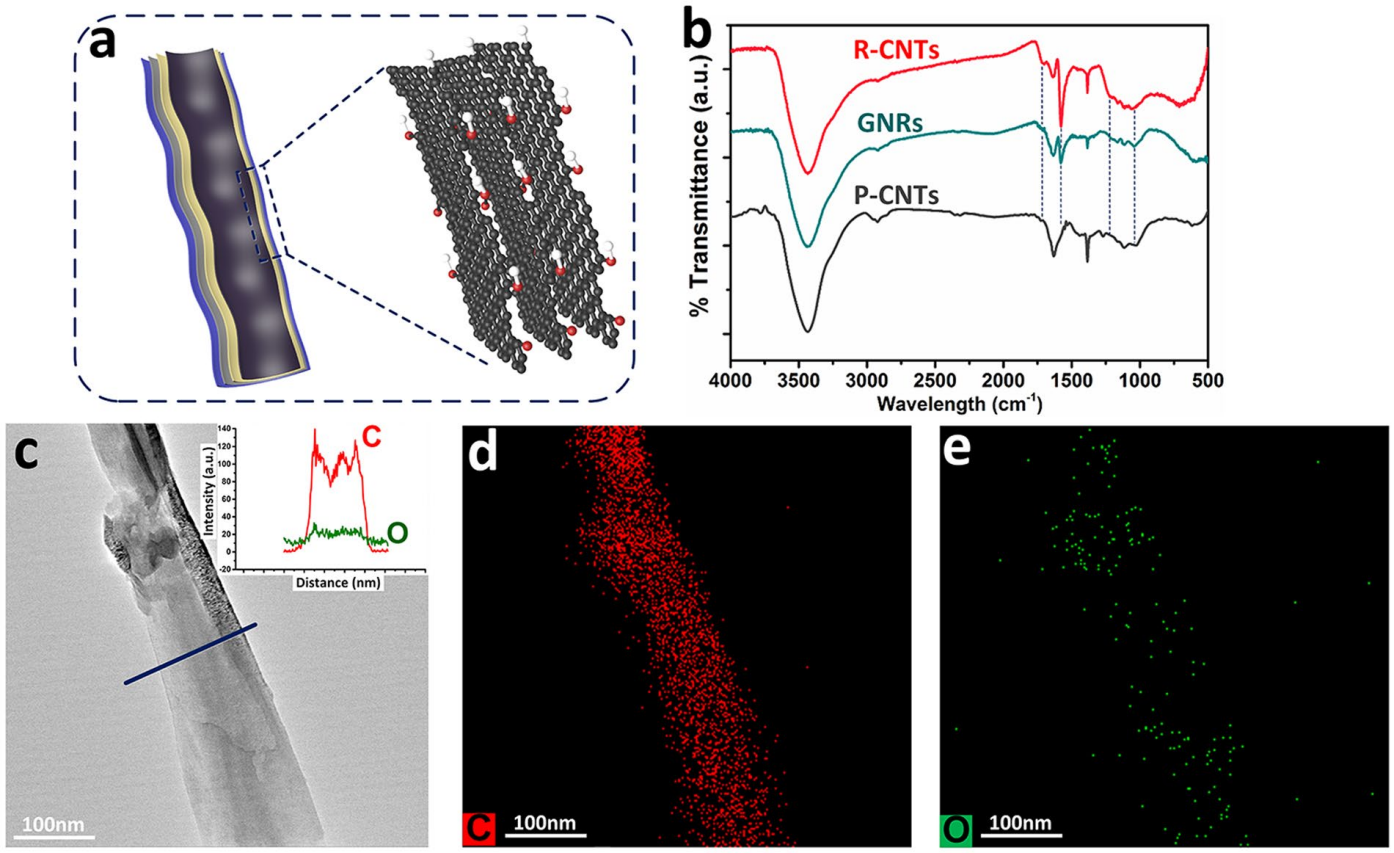
(**a**) Scheme depicts the edge-selective dispersion of C–O in the nanobelts. (**b**) FTIR spectra of P-CNTs, GNRs and R-CNTs. (**c**) TEM image of a fully-opened graphite nanobelt, inset shows the line scans of O and C elements. (**d**,**e**) The corresponding C and O elemental mapping.

R-CNTs are well-dispersed in solvents because of the covalent grafting of active oxygenated groups (*e*.*g*. epoxy, hydroxyl and carboxyl groups) on the surface^3,13^. GNRs are also hydrophilic and dispersible in NMP owing to the insertion of C–O along the lateral walls^29^. It is noteworthy that both the species and quantity of functional groups on GNRs are less than those on R-CNTs. This is attributed to distinct oxidation regimes between gas-phase and liquid-phase treatments. Longitudinal splitting of CNTs *via* HNO_3_ steaming follows an edge-selective oxidation mechanism, as illustrated by the schematic diagram of Fig. 3a. The oxidation of GNRs occurs preferentially in the opened lateral planes. As a result, this splitting process is evidenced to be nonaggressive, which accounts for the minimized oxidation degree and well-preserved sp^2^ framework in the basal planes^29^. At variance, the refluxing process follows non-selective oxidation steps in terms of intercalation, substitution and unravelling, which occurs on both the remaining sidewalls and exfoliated graphene basal planes.

The difference in surface functionalities is verified by the FTIR spectra (Fig. 3b). Only weak and shallow C–O characteristic peaks (1050 cm^−1^, 1250 cm^−1^ and 1570 cm^−1^ assigned to epoxide, hydroxyl, and phenolic groups, respectively) are presented in GNRs. In contrast, in addition to more intensified C–O characteristic peaks, an obvious O–C = O characteristic vibration peak situated at 1720 cm^−1^ is detected in R-CNTs. Moreover, both the EDS elemental mappings (Fig. 3c–e) and line scans (Fig. 3c inset) further support a relative low density of oxygen species, which are more amenable to an appearance at the margins than in the basal planes of the unrolled nanoribbons.

**Figure 4:**
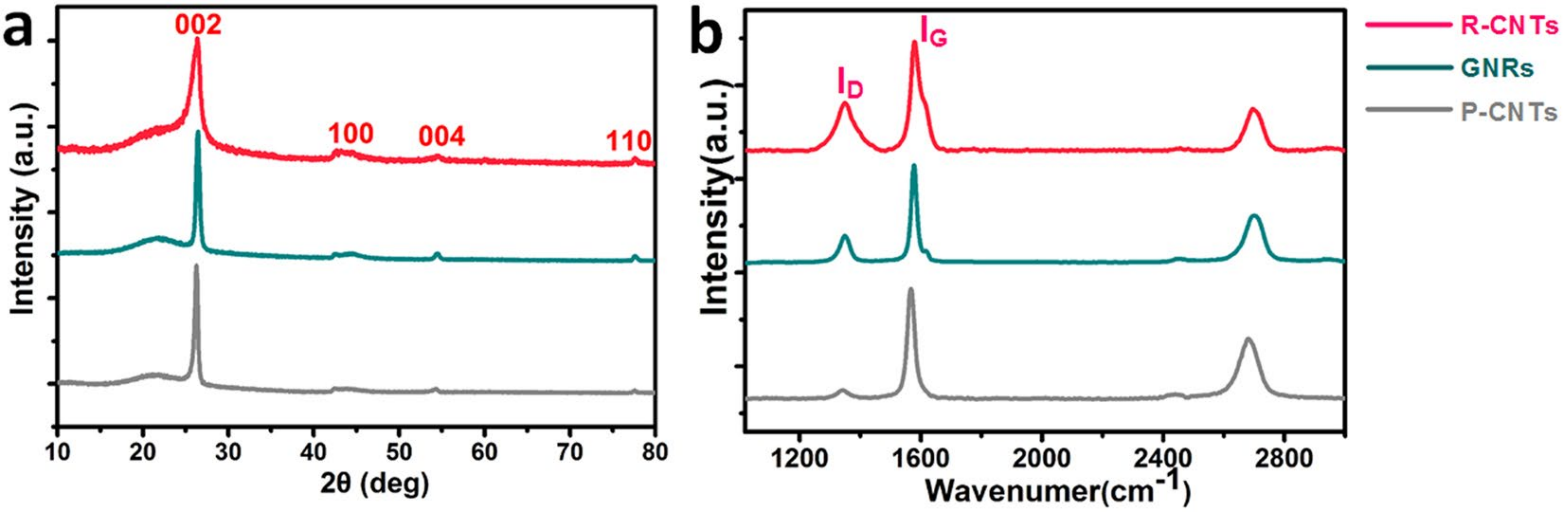
(**a**) XRD and (**b**) Raman spectra of P-CNTs, GNRs and R-CNTs. The data are normalized.

The different levels of covalent grafting between GNRs and R-CNTs are further reflected on the structural integration and crystalline quality. Figure 4a exhibits the XRD patterns of P-CNTs, GNRs and R-CNTs. The existence of intense (002) peak at ~26° implies a highly-ordered graphitic lattice of P-CNTs. The XRD profile of GNRs matches well with the corresponding diffraction pattern of P-CNTs, confirming their well-preserved crystalline structure. In contrast, the broadened peak of R-CNTs near 2θ = 26° reveals an existence of local disorder between adjacent nanotube walls^31^. Raman spectroscopies (Fig. 4b) enable us to monitor structural changes and any generated defects. The relative degree of structural defects and disorder in carboneous materials are usually evaluated by analysis of the D (~1350 cm^−1^) to G (~1580 cm^−1^) peak intensity ratio (**I**_**D**_ **/I**_**G**_). The extracted I_D_/I_G_ ratio of P-CNTs is 0.08, which is an extremely low value, confirming the highly crystalline nature of P-CNTs. In contrast to the high I_D_/I_G_ ratios of R-CNTs (~0.45), GNRs show moderately increased I_D_/I_G_ ratios (~0.27) due to the cleavage of side-walls, implying a relatively lower degree of disorder. The I_D_/I_G_ ratio of GNRs is much smaller than those of nanocarbons fabricated by commonly used liquid-phase oxidation methods (*e.g*. acid reflux and the Hummers method), as shown in Table 1. In addition, the I_D_/I_G_ ratio of GNRs is comparable with those of graphene^32^ and CNTs^33^ with well-defined structures, which unambiguously proves their high crystalline quality.

**Table 1:** I_D_/I_G_ ratios of GNRs, R-CNTs and other nanocarbons fabricated by different methods.

Nanocarbon Type	I_D_/I_G_	Fabrication method	Ref.
Graphene oxide	1.47	Liquid-phase oxidation	32
Graphene	0.24–0.4	Mechanical exfoliation	32
MWCNT	0.65	CVD	33
MWCNT	0.3	CVD	44
Graphene nanoribbon	1.6	Liquid-phase oxidation	44
Graphene nanoribbon	0.82–1.1	Liquid-phase oxidation	26
Graphene nanoribbon	1.5–2	Liquid-phase oxidation	27
R-CNT	0.65	Liquid-phase oxidation	This work
GNR	0.27	Gas-phase oxidation	This work

### GNRs as highly-efficient polymer reinforcement

**Figure 5:**
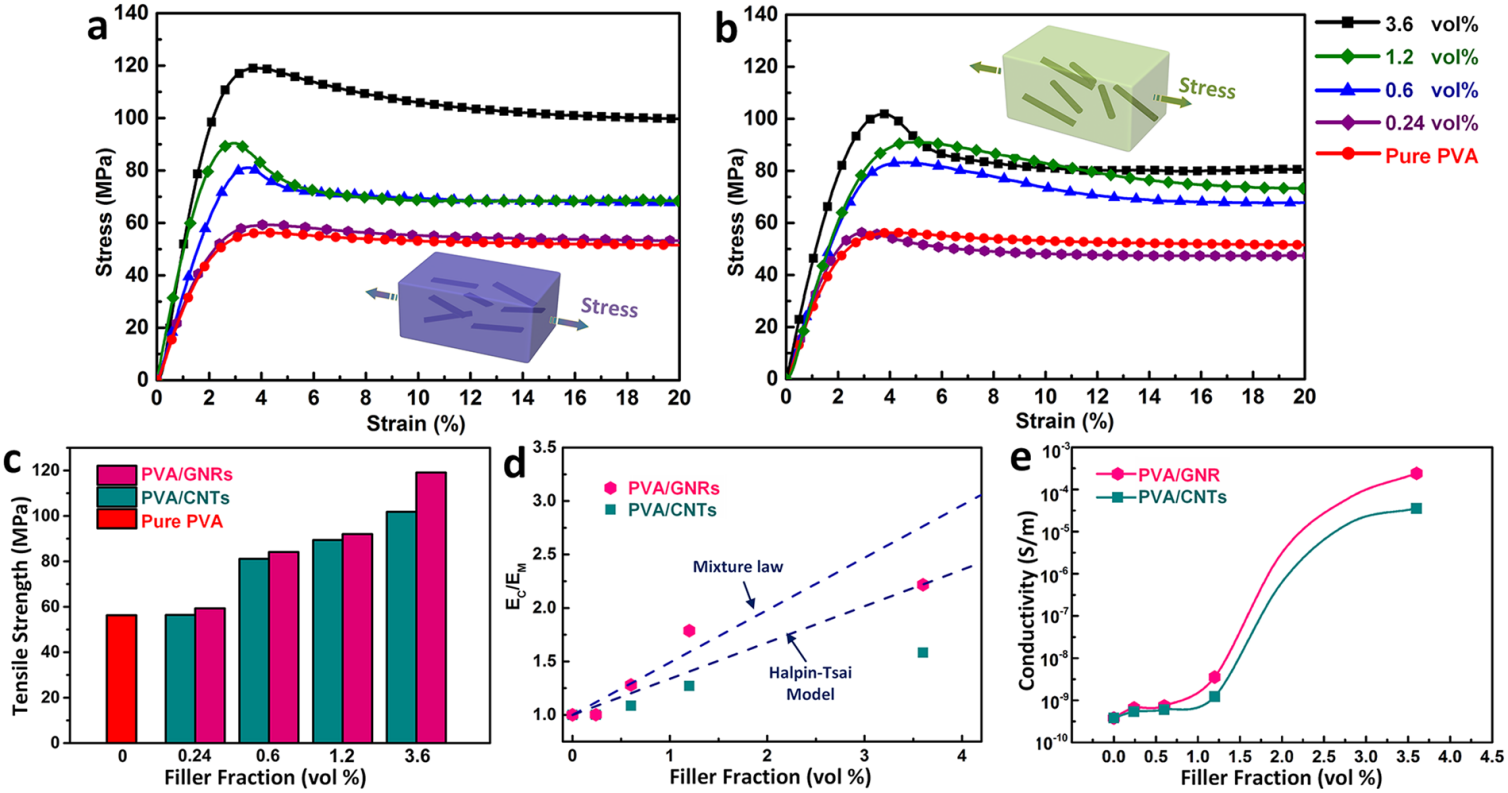
Typical stress-strain curves of PVA filled with different amounts (0/0.24/0.6/1.2/3.6 vol.%) of: (**a**) GNRs and (**b**) R-CNTs. Insets show the corresponding schematic illustrations for the load-bearing of PVA/GNRs and PVA/CNTs. (**c**) Comparison of tensile strength values of the composite films. (**d**) Normalized Young’s modulus of PVA-based composites (E_c_/E_m_) plotted *vs*. nanofiller volume fraction. The experimental data are compared with theoretical predictions from the mixture rule and Halpin-Tsai model. (**e**) Plots of composite conductivities *vs*. nanofiller volume fraction.

It is known that nanofiller solubility, dispersion, stress transfer^3^ and morphology (including the size, shape and the number of chemical defects presented)^34–36^ must all be maximized to reach optimum properties. The combination of unique geometry, surface functionality, high aspect ratio, well-preserved structural integrity and robust electrical property of the obtained GNRs motivates us to employ them as enhancers, with expectation to improve both the mechanical and electrical performances of pure polymers. We fabricate PVA-based composite films (*i*.*e*. PVA/GNRs and PVA/CNTs) at different additive loadings (0–3.6 vol.%) using a solution casting method. As shown in Fig. 5a–c, the mechanical properties for pure PVA are enhanced by incorporating either GNRs or R-CNTs, with an enhancement proportional to the filler fraction. Noteworthy, thanks to the flat shape and robust physicochemical properties, GNRs play a more significant reinforcement role than R-CNTs at the same filler concentration. Specifically, PVA/GNRs (3.6 vol.%) show a tensile strength of 119.1 MPa and Young’s modulus of 5.3 GPa, which are ~17% and ~32.5% higher than those of PVA/CNTs (101.8 MPa and 4 GPa), respectively.

While CNTs are less effective at reinforcing PVA composites, splitting and unpeeling them into GNRs result in a notable improvement, owing to mainly four factors as follows: (i) a substantial retaining of graphitic structure and intrinsic physical properties; (ii) an effective utilization of the inner nanotube layers and thus a pronounced increase of the surface area (*i*.*e*. interfacial contact area); (iii) it is easier for polymer macromolecules to adhere and mechanically interlock to a planar nanofiller than a tubular one^19^; (iv) a stronger cohesion force with PVA matrix owing to increased interfacial contact area. This improvement is verified by a more reasonable agreement between experimental data and theoretical predictions of Young’s modulus of PVA/GNR composites compared to that of PVA/CNTs, as depicted in Fig. 5d. The elastic properties of the composites are predicted by the well-established Halpin-Tsai model^19,37,38^ and mixture law. For randomly oriented fiber fillers in a polymer matrix, the composite modulus ${E}_{c}$ is given by:

1 $${E}_{c}={E}_{m}[\frac{3}{8}(\frac{\xi {\eta }_{l}{V}_{f}+1}{1-\xi {\eta }_{l}{V}_{f}})+\frac{5}{8}(\frac{2\,\xi {\eta }_{w}{V}_{f}+1}{1-\xi {\eta }_{w}{V}_{f}})]$$

2 $${\eta }_{l}=\frac{\frac{{E}_{f}}{{E}_{m}}-1}{\frac{{E}_{f}}{{E}_{m}}+\xi }$$

3 $${\eta }_{w}=\frac{\frac{{E}_{f}}{{E}_{m}}-1}{\frac{{E}_{f}}{{E}_{m}}+2}$$

4 $${\rm{For}}\,{\rm{GNRs}},\xi =\frac{w+l}{t}$$

5 $${\rm{For}}\,R-\mathrm{CNTs},\xi =2\frac{l}{d}$$

where ${E}_{m}$ are the modulus of the PVA matrix, ${V}_{f}$ and ${E}_{f}$ are the volume fraction and modulus of fillers, *d* is the diameter of P-CNTs, *w*, *l*, and *t* are the width, length and thickness of GNRs, respectively. At the same additive fraction, GNRs contribute to a more efficient modulus enhancement than CNTs. Given that the fabrication methodology and the dispersion, orientation, length of inclusions are identical for both PVA/GNRs and PVA/CNTs, we conclude that the difference in load-transfer efficiencies should be exclusively correlated to the morphological modification.

Furthermore, the electrical conductivity of PVA/GNRs is also enhanced as compared to that of PVA/CNTs (Fig. 5e). The direct-current conductivity of bulk PVA/GNR films (3.6 vol.%) is measured as 2.4 × 10^−4^ S m^−1^, which is significantly higher as compared to that of PVA/CNTs (3.5 × 10^−5^ S m^−1^). As previously reported^29^, the intrinsic conductivity of individual GNRs is deduced as 2.2 × 10^4^ S m^−1^ from conducting-probe AFM measurements, which is almost an order of magnitude higher than that of R-CNTs (2.4 × 10^3^ S m^−1^). This directly collaborates a better structural integrity of GNRs, and hence, they will offer a better conducting network, which is responsible for the enhanced electrical conductivity of PVA/GNRs over that of PVA/CNTs. Another reason for the superior electrical conductivity of PVA/GNRs may be the increased interfacial contact area, which contributes to more robust charge transfer path. The pure PVA films are insulators but the incorporation of nanofillers results in highly conductive films. Therefore, these GNRs show high efficiency in reinforcing polymers, as mentioned above, enabling outstanding polymeric composites with attractive and promising multi-functionalities.

### The Role of Structural Modification

**Figure 6:**
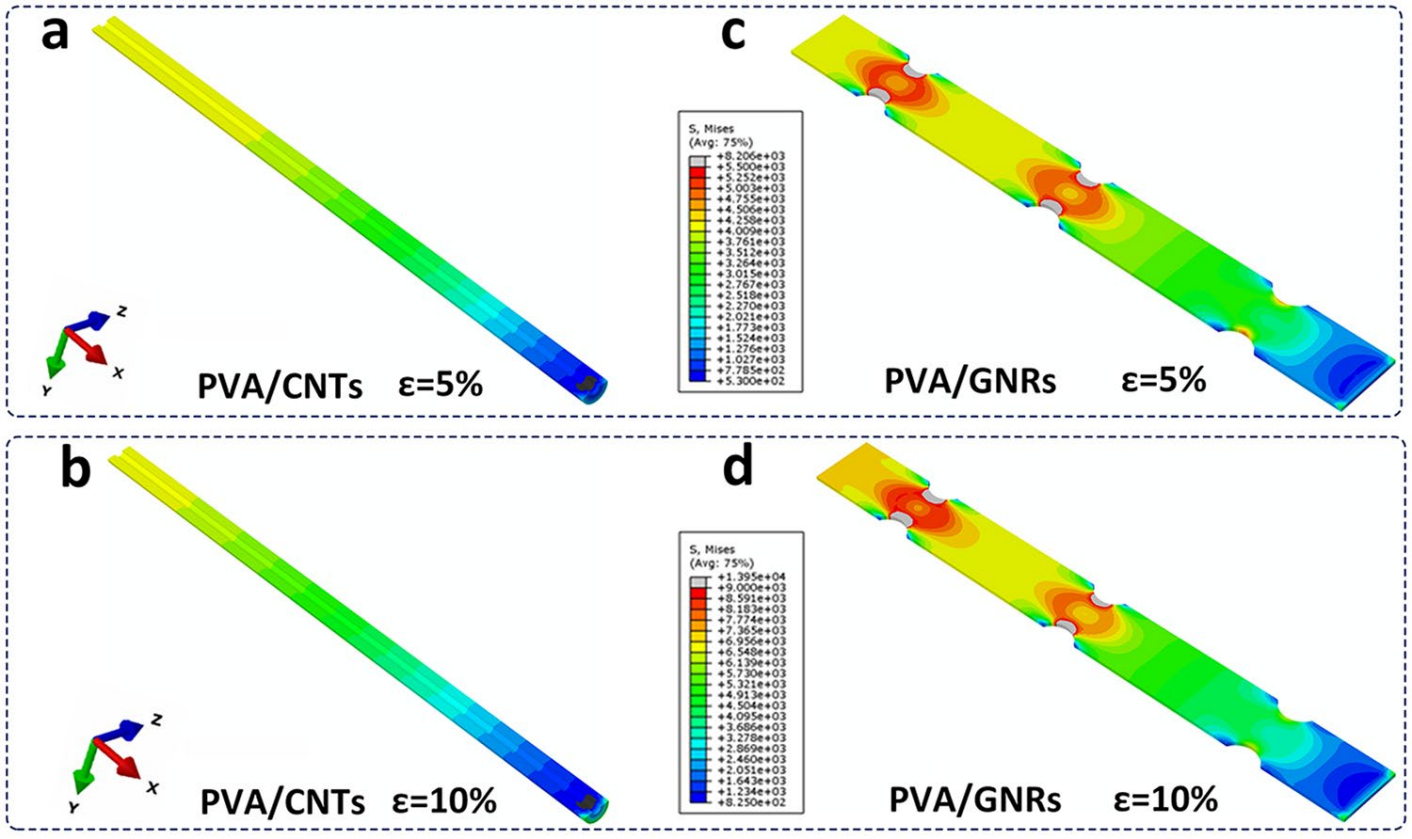
The extracted contour plots of von Mises stress distribution on (**a**,**b**) R-CNTs and (**c**,**d**) GNRs. The stress distributions correspond to tension strains ($\varepsilon$) of 5% (**a** and **c**) and 10% (**b** and **d**), respectively. The tensile loading direction is along the X axis, and only half of the nanofiller model is displayed.

Numerical simulations are further performed using a commercial finite-element method (**FEM**) software (ABAQUS) to get more insights into the effect of morphological modification on the stress distribution, load-carrying capacity of CNTs and the overall mechanical response of the bulk composites. Figure 6 represents a comparison of von Mises stress distribution on a single CNT and an “opened GNR from the CNT” obtained by FEM simulations. At a tensile strain ($\varepsilon$) of either 5% or 10%, the magnitude of stress on the nanoribbons is notably larger than that on the nanotubes. As mentioned before, the SSA of GNRs is 1.6 times as large as that of R-CNTs. Thereby, it is distinguished that GNRs may enable a more prominent stress bearing capacity (*i*.*e*., load-transfer from matrix to inclusion, defined as $F=\oiint \sigma ds$)^16^ than R-CNTs, owing to strong interfacial interaction during composite deformation. Molecular dynamics simulations have also proven the poor resistance to sliding separation of CNTs as an outcome of the smooth surface, but nanoplatelets can resist normal separation and have an interfacial normal strength that is higher than the shear strength^36^.

For polymer nanocomposites, the interfacial shear strength is typically determined by the surface chemical interaction with the matrix and the fiber surface area that contributes to effective load transfer^39^. Given that the interfacial cohesion characteristics of PVA/GNRs is identical to that of PVA/CNTs, the discrepancy of mechanical properties between PVA/GNRs and PVA/CNTs should be exclusively ascribed to the nanoengineering of filler morphologies. When serving as nanofillers, their size, dimension and geometry substantially determine the interfacial configuration, load-transfer characteristic and consequently the overall mechanical performance of composites at the macroscale^16^. It is easier for PVA chains to adhere to a flat nanofiller with a sheet or ribbon-like geometry^19^, and thus GNRs with platelet shape can act as more effective load-carrying units than the tubular CNTs. In addition, the nanoribbons are beneficial to a more robust polymer-nanocarbon interaction by mechanical interlocking due to their rough and irregular edges (see Fig. 2c), which is different with the easy interfacial slide and debonding of the smooth tubular nanotubes^15^. Consequently, splitting CNTs into GNRs leads to a significant improvement of load-transfer efficiency.

### Fracture Modes of GNRs and CNTs

The influence of morphological change of nanofillers on the performances of composites can be further clarified using the well-established shear-lag theory. This theory states that load-transfer from matrix to the enhancer occurs *via* the shear stress generated at the polymer-inclusion interface^18,34^. Accordingly, there is an critical length ${l}_{c}$ for the reinforcement to carry a maximum stress at its midpoint and maximize the composite strength^4,40^:

6 $${\rm{For}}\,R-\mathrm{CNTs},{l}_{c}=\frac{{\sigma }_{f}d}{2{\tau }_{m}}[1-\frac{{d}_{i}^{2}}{{d}^{2}}]$$

7 $${\rm{For}}\,{\rm{GNRs}},{l}_{c}=\frac{{\sigma }_{f}\times w\times l\times t}{2{\tau }_{m}\times (w+l)\times t}$$

where ${\tau }_{m}$ is the interfacial shear stress (2–400 MPa for polymer-nancocarbon systems^40^), ${\sigma }_{f}$ is the tensile strength of nanofillers, and *d*_*i*_ is the internal diameter of R-CNTs. Nanofillers fail under the fracture mode and play a prominent load-carrying role when *l* > *l*_*c*_, whereas they can be pulled out when *l* > *l*_*c*_. For GNRs, their additional contact area will enable a more distinguished interfacial interaction with PVA (in the forms of mechanical interlocking, van der Walls force and hydrogen bonding) and thus a reasonably increased interfacial shear stress of PVA/GNRs than that of PVA/CNTs. In addition, GNRs with platelet shape and rough edges is less prone to slipping than nanotubes. Thereby, ${\tau }_{m}$ is set as 50 MPa and 25 MPa for GNRs and R-CNTs, and the corresponding ${l}_{c}$ is deduced as 5.8 μm and 11.7 μm for GNRs and R-CNTs, respectively. Accordingly, GNRs correspond to a fracture mode ($l=6\,\mu m > {l}_{c}=5.8\,\mu m$) whereas CNTs follow a pull-out mode ($l < {l}_{c}$).

**Figure 7:**
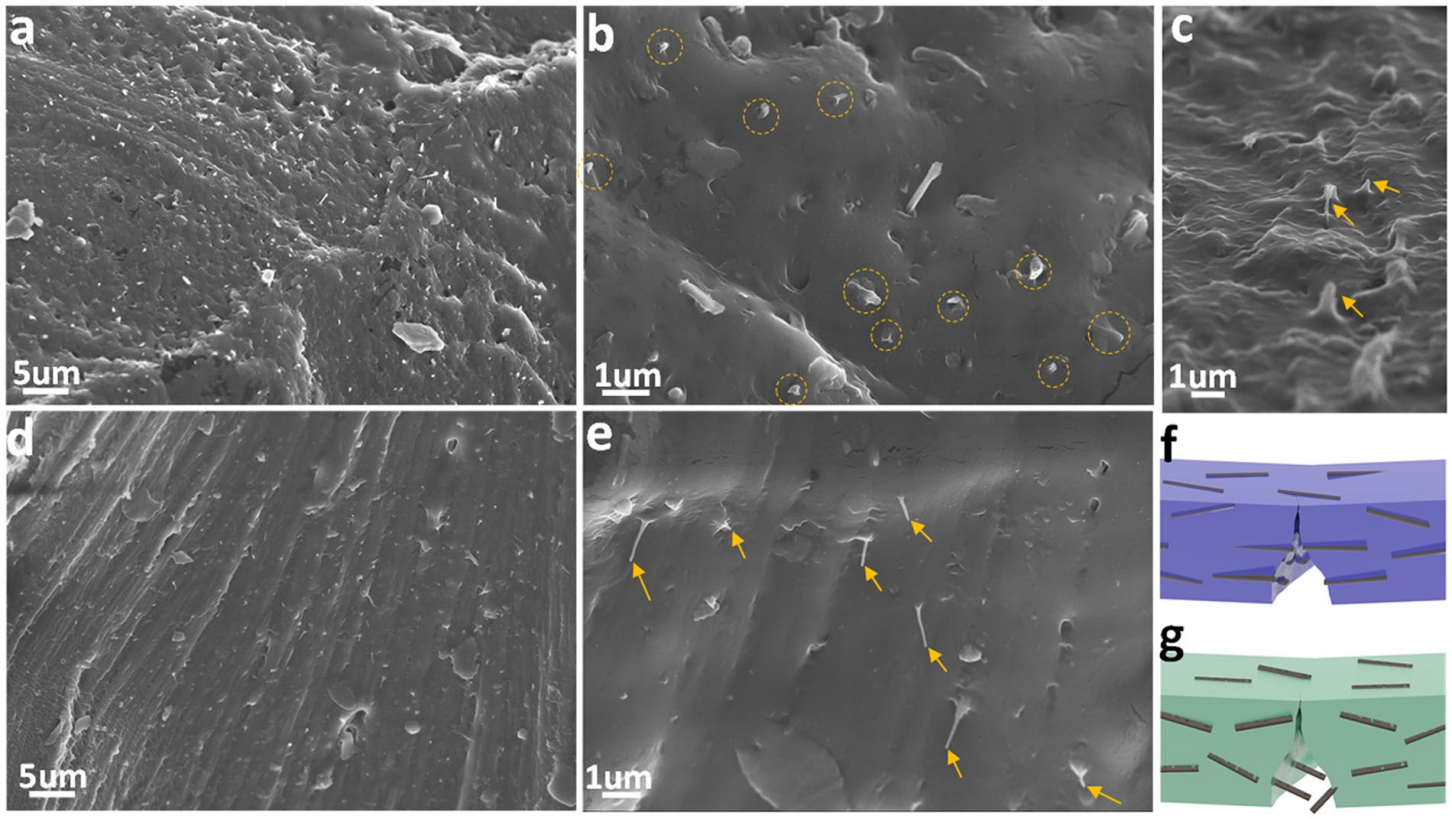
SEM micrographs showing the fracture surfaces of (**a**,**c**) PVA/GNRs and (**d**,**e**) PVA/CNTs. The filler fraction is 3.6 vol.%. It is observed that both the GNRs and R-CNTs are uniformly dispersed in the PVA substrate, indicated by the circles and arrows. GNRs are fractured and well-embedded in the substrate whereas R-CNTs are obviously pulled out. Schematic illustrations showing the corresponding fracture mechanism of (**f**) GNRs and (**g**) CNTs in PVA environment.

Fractured surfaces (Fig. 7) indicate that both GNRs and R-CNTs are distributed uniformly inside the PVA matrix without obvious aggregation. The fracture patterns demonstrate that GNRs are fractured with very short protruding length (<100 nm), and they are well-embedded in the polymer substrate (Fig. 7a,b). Moreover, the accompanied deformation of neighbouring matrix suggests a considerable energy dissipation ((Fig. 7c)^28^. The failure mechanism of GNRs in polymer matrix corresponds to a ‘bridging-fracture’ manner. In contrast, R-CNTs are evidently pulled out from the matrix (Fig. 7d,e), suggesting an interfacial slippage and weak load-transfer efficiency^41^. These failure modes agree well with the aforementioned shear-lag analysis, clearly proving that GNRs effectively strengthen the composite through the conventional load-transfer mechanism^42^.

It is noteworthy that there is a jump of both mechanical and electrical properties for the PVA/GNR system, as the addictive loading increases from 0.6/1.2 vol.% to 3.6 vol.%. This can be ascribed to a threshold effect. The electrical percolation phenomenon is well-defined for nanocarbon in polymer composites^6^. This percolation effect is also valid to rationalize the mechanical behaviors. At GNR concentration below 1.2 vol.%, the strength improvement mainly originates from the load bearing of GNRs. However, when GNR concentration exceeds ~ 3.6 vol.%, the strength improvement is attributed to not only the load-bearing effect but also the matrix hardening. We believe that every individual GNR expresses a stress-affected zone to the neighbouring polymer matrix^28^, and there is a critical filler fraction for these micro-zones to interconnect and deliver a jump of strength. This is further supported by the different fracture morphologies, in which, the matrices are smooth after deformation at 0.6/1.2 vol.% GNRs (Figure S2a,b) whereas the matrix undergoes severe deformation and shows considerable roughness at 3.6 vol.% GNRs (Figure S2c).

## Conclusion

In conclusion, we present here a configuration design of carbon nanofillers *via* longitudinal splitting MWCNTs to form high-quality GNRs, which provides an avenue to simultaneously modulate the mechanical behavior and electrical properties of polymer composites. We have demonstrated that high-quality GNRs are more effective reinforcements than unmodified MWCNTs. GNRs are conducive to strong interfacial cohesion, load bearing and conducive network, owing to their quasi-1D dimension, straight and planar geometry, high aspect-ratio, superb intrinsic strength and remarkable conductance. As a result, the tensile strength, Young’s modulus and electrical conductivity of PVA/GNR composite at a filling concentration of 3.6 vol.% approach 119.1 MPa, 5.3 GPa and 2.4 × 10^−4^ S m^−1^, showing increases of 17%, 32.5% and 5.9 folds respectively, as compared to PVA/CNTs. The planar geometry renders GNRs with a better load-sharing efficiency than their unmodified counterparts, which is rationalized by the shear-lag model, FEM simulations and fractography analysis. This study sheds light on the nanofiller design for advanced composites. The utilization of nanoribbons as composite fillers instead of nanotubes and such concept of structural design are versatile, which can be extended to other hybrid systems (*e.g*. other polymer, metal and ceramic matrices) for developing of high-performance composites with multi-functionalities.

## Experimental Section

### Raw Materialsh

P-CNTs used in this study were purchased from Showa Denko Group (Tokyo, Japan). They were fabricated by catalytic chemical vapor deposition process followed by high-temperature annealing (>2800 °C). P-CNTs have a length of ~6 μm and an average diameter of 120 nm. PVA (degree of polymerization, ~1799; hydrolysis degree, 98–99%) and NMP (>99.0 wt.%) were purchased from Aladdin Industrial Corp. (Shanghai, China). The concentrated sulfuric acid (H_2_SO_4_, 97 wt.%) and nitric acid (HNO_3_, 61 wt.%) were of analytical grade and provided by Sinopharm Chemical Regent Co., LTD (Shanghai, China).

### Preparation of GNRs

GNRs were fabricated by a gas-phase treatment method *via* steaming P-CNTs with HNO_3_ vapor. Additional details regarding the apparatus and processes are available in previous literatures^29,43^. Briefly, as-received CNTs (300 mg) were loaded on a self-designed glass steamer with a porous silica griddle. The steamer was then placed into a 50 mL Teflon vessel, at the bottom of which 3 mL HNO_3_ was loaded. Subsequently, the Teflon vessel was encapsulated in an autoclave and kept at 220 °C for 5 h in a furnace. The concentrated HNO_3_ was volatilized to steam and reacted with the solid P-CNTs in this high-temperature and high-pressure environment. Finally, the steamed CNTs were *in-situ* washed for several times, followed by vacuum drying at 60 °C for 24 h. The resulting product was rinsed, filtered and dried for further characterization or application as PVA reinforcements.

### Preparation of R-CNTs

For comparison, P-CNTs were also treated by the commonly used acid reflux method with H_2_SO_4_/HNO_3_ mixtures (v/v = 3:1) to facilitate their dispersion in solvents. In general, P-CNTs (100 mg) were dispersed in liquid acid (100 ml) in a 200 ml round bottom flask equipped with a condenser. Then the dispersion was heated and refluxed at 100 °C for a dwelling time of 20 min. The resultants were washed up to neutral pH, filtered, and dried in vacuum at 60 °C for 24 h.

### Solution Mixing of Composite Films

As-synthesized GNRs were re-dispersed in NMP (100 mL of NMP per 100 mg of GNRs) by tip sonication at 200 W (GN-1000Y, Guning Instrument Co., Ltd, Shanghai, China) for 24 h in an ice bath. The mild sonication treatment was also beneficial to a further exfoliation of the thick layers of GNRs. Meanwhile, PVA (1.67 g) was dissolved in *D.I*. water (15 mL) at 90 °C for 1 h to give a 10 wt.% solution. Then, the two dispersions were mixed together by stirring for 2 h followed by 0.5 h of sonication. The homogenous PVA/GNR mixture was then poured into a culture dish with a diameter of 8 cm. Finally, PVA/GNR composite films were obtained after removing the solvents in an oven at 60 °C for 48 h. For comparison, PVA/CNT composite films were also prepared using the same methodology. PVA/GNR and PVA/CNT composites with varying volume fractions (0, 0.24, 0.6, 1.2 and 3.6 vol.%) were fabricated by adjusting the amount of GNRs or R-CNTs.

### Characterization

The fracture morphologies of composite films were characterized using a field-emission scanning electron microscope (**SEM**, S-4800, HITACHI). A transmission electron microscopy (**TEM**) and high resolution TEM (**HRTEM**, JEM 2100 F, JEOL) equipped with an energy dispersive spectrometer (**EDS**) was employed to obtain information on the nanostructure of GNRs and R-CNTs, operating at 200 KV. The structural characteristics were analyzed using a Raman spectroscopy (SENTERRA R200, Renishaw) and a powder X-ray diffraction (**XRD**, Ultima IV, Rigaku). Raman spectroscopies were collected using an excitation wavelength of 532 nm under a 200 s acquisition time. XRD measurements were carried out using Cu Kα (λ = 1.5406 Å) radiation operating at 35 kV and 200 mA. The data were collected for a 2θ angle ranging from 10° to 80° with a scan rate of 5° min^−1^ and a step size of 0.02°.

Fourier transform infrared spectroscopy (**FTIR**, Nicolet 6700, ThermoFisher) was used to analyze the surface chemistry. FTIR spectra were obtained using KBr discs with a scan range of 400−4000 cm^−1^ and a mean signal of 64 scans at a resolution of 4 cm^−1^. The topographic imaging was done by tapping mode at room temperature using an atomic force microscope system (**AFM**, D8 Advance, Bruker). Samples were prepared by casting onto a clean Si/SiO_2_ substrate. The SSA was monitored by means of N_2_ adsorption-desorption at 77 K in liquid nitrogen (ASAP 2020, Micrometritics). The SSAs were calculated using Brunauer-Emmer-Teller (**BET**) model.

### Mechanical and Electrical Tests

PVA-based composite films with a thickness of 0.3–0.5 mm were cut into strips of 48 mm × 12 mm using a razor blade. Uniaxial tensile mode tests with an initial gauge length of 18 mm were implemented using a Zwick/Roell Z020 system at a crosshead speed of 4 mm min ^−1^ at room temperature (~22 °C). At least five strips were tested for each composite type. Electrical conductivities of PVA/GNR and PVA/CNT composites were measured by standard four-probe technique (RTS-8, 4probes Tech, Shenzhen, China). Silver paste (SPI Supplies, USA) electrodes of 5 mm long were realized on the specimens.

### FEM Simulation

FEM simulations were conducted using a commercial ABAQUS software (version 6.14). The matrix and nanofillers were meshed using eight-node linear brick elements (C3D8 in ABAQUS), whereas the interface layers were meshed using eight-node 3D cohesive elements (COH3D8 in ABAQUS). The representative volume element is 8 μm in length, 0.552 μm and 0.548 μm in diameter for PVA/GNRs and PVA/CNTs, respectively. The GNR model has a strip-like shape with 6 μm in length, 0.3 μm in width and 40 nm in thickness, and six arc-shaped notches were introduced on both sides to describe the irregular edges. The CNT model has a tubular shape with a length of 6 μm, an outside diameter of 120 nm and an inside diameter of 40 nm. The specimens were stretched on both sides along the X direction. The volume fraction was set as 3.6%.

## Electronic supplementary material

Supplementary Information
