# Supplementary material for: Simultaneously improving the mechanical and electrical properties of poly(vinyl alcohol) composites by high-quality graphitic nanoribbons
Source: Sci Rep. 2017 Dec 7;7:17137. doi: 10.1038/s41598-017-17365-3 (PMC5719439; doi:10.1038/s41598-017-17365-3)
Supplement: Supplementary file 1 — Supplementary Information [file 41598_2017_17365_MOESM1_ESM.pdf]

Simultaneously improving the mechanical and electrical properties of poly(vinyl alcohol) composites by high-quality graphitic nanoribbons

Ming Yang[1], Lin Weng[1], Hanxing Zhu[2], Fan Zhang[1], Tongxiang Fan[1,*], Di Zhang[1]

[1]State Key Laboratory of Metal Matrix Composites, Shanghai Jiao Tong University, 800 Dongchuan Road, Shanghai 200240, PR China

[2]School of Engineering, Cardiff University, Cardiff, CF24 3AA, UK

*T. Fan (txfan@sjtu.edu.cn)

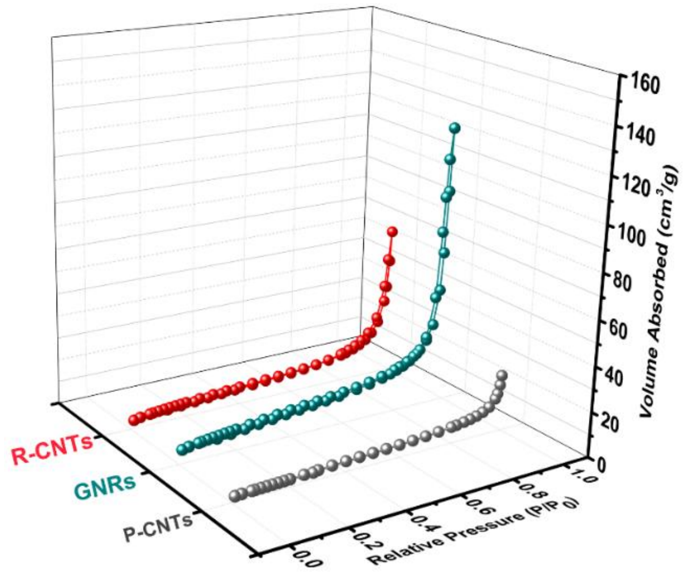

Figure S1. N_2 absorption-desorption isotherms of P-CNTs, GNRs and R-CNTs. The derived BET surface areas are 11.7 m^2 g^{-1}, 35.8 m^2 g^{-1} and 22.6 m^2 g^{-1}, respectively.

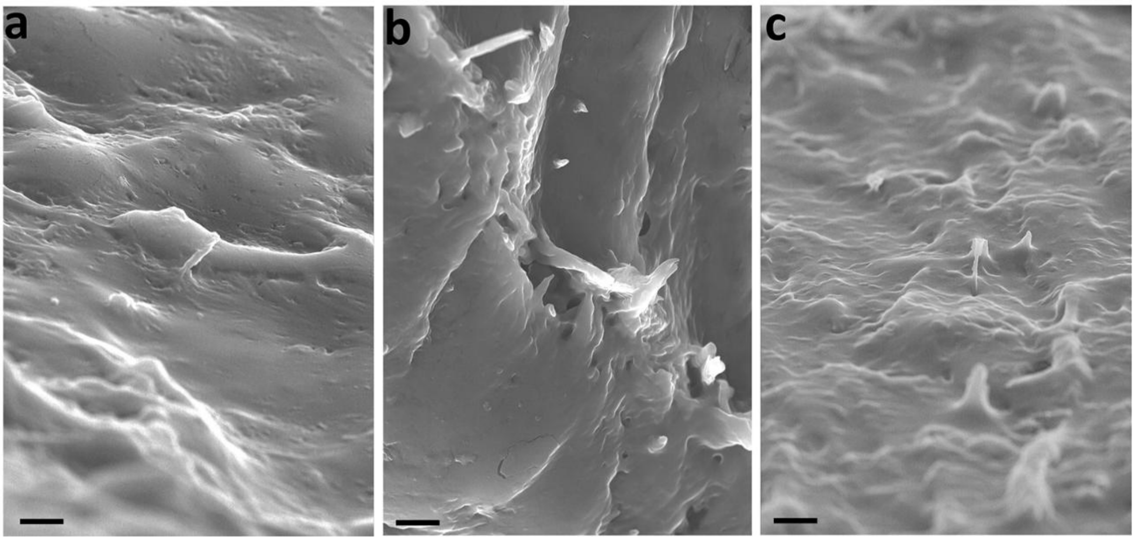

Figure S2. SEM images showing the fracture surfaces of PVA/GNRs with GNR fraction of (a) 0.6 vol.%, (b) 1.2 vol.% and (c) 3.6 vol.%. The scale bars are 0.5 μm.
